# Clinical Outcomes After Ultrathin Descemet Stripping Automated Endothelial Keratoplasty Versus Descemet Membrane Endothelial Keratoplasty for Fuchs Endothelial Corneal Dystrophy: A Systematic Review and Meta-Analysis

**DOI:** 10.7759/cureus.105062

**Published:** 2026-03-11

**Authors:** Dimitra Katerini, Anastasia Tsiogka, Konstantina Koulotsiou, Konstantinos Droutsas

**Affiliations:** 1 Ophthalmology Department, Venizeleio General Hospital of Heraklion, Heraklion, GRC; 2 Ophthalmology Department, National and Kapodistrian University of Athens, General Hospital of Athens "G. Gennimatas", Athens, GRC; 3 Ophthalmology Department, General Hospital of Athens "G. Gennimatas", Athens, GRC; 4 First Ophthalmology Department, National and Kapodistrian University of Athens, General Hospital of Athens "G. Gennimatas", Athens, GRC

**Keywords:** corneal transplantation, dmek, donor cornea, fuchs endothelial corneal dystrophy, ut-dsaek

## Abstract

In Fuchs endothelial dystrophy (FED), the corneal endothelial layer undergoes progressive degenerative changes leading to a reduction in endothelial cell count and subsequent visual impairment. Descemet membrane endothelial keratoplasty (DMEK) and ultrathin Descemet stripping automated endothelial keratoplasty (UT-DSAEK) are two established surgical approaches for the management of FED. This meta-analysis aims to compare postoperative clinical outcomes between DMEK and UT-DSAEK in adult patients with FED. A literature search was conducted in major peer-reviewed electronic databases. Best-corrected visual acuity (BCVA) served as the primary outcome, while endothelial cell density (ECD) and postoperative complications were evaluated as secondary outcomes. Compared with UT-DSAEK, DMEK demonstrated a tendency toward better BCVA at one year, although it was also associated with a higher likelihood of requiring re-bubbling. No clear differences were observed between the two techniques regarding ECD at 12 months, while complication rates varied between studies. Overall, DMEK showed more favorable visual outcomes, whereas UT-DSAEK presented a lower complication burden. Although complications following DMEK are generally manageable, some may necessitate additional surgical procedures. Both techniques remain effective options for corneal surgeons treating patients with FED.

## Introduction and background

More than a century has passed since the first successful penetrating keratoplasty (PK) was performed by Eduard Zirm in 1905. Several decades later, advances in corneal transplantation led to the development of endothelial keratoplasty (EK), first introduced by Melles in 1999. Since then, multiple modifications of EK techniques have been developed in order to improve visual outcomes while reducing postoperative complications and graft rejection rates [1].

EK has gradually replaced PK as the preferred surgical approach for endothelial dysfunction, as it selectively replaces the diseased endothelium while preserving most of the recipient corneal structure. Compared with PK, EK techniques are associated with faster visual recovery, less postoperative astigmatism, and improved wound stability [2,3]. In addition, the reduced amount of transplanted tissue may contribute to a lower risk of immune-mediated rejection compared with full-thickness keratoplasty [4].

Among EK procedures, Descemet membrane endothelial keratoplasty (DMEK) involves transplantation of isolated donor Descemet membrane and endothelium, which are inserted, unfolded, and positioned within the recipient cornea. In contrast, Descemet stripping automated endothelial keratoplasty (DSAEK) utilizes a donor graft that includes a thin layer of posterior stromal tissue. Ultrathin DSAEK (UT-DSAEK), typically defined by graft thickness below 130 μm, was introduced to improve visual outcomes compared with conventional DSAEK while maintaining greater surgical stability [5].

Fuchs endothelial corneal dystrophy (FED) is one of the most common indications for EK worldwide. The disease is characterized by progressive degeneration of corneal endothelial cells, leading to reduced endothelial cell density (ECD) and eventual corneal edema. In healthy adults, ECD ranges between approximately 2500 and 3000 cells/mm² and gradually decreases with age. When ECD falls below critical levels, corneal decompensation may occur, resulting in visual impairment and the need for corneal transplantation [6,7]. Patients with FED typically experience progressive blurred vision, glare, and reduced contrast sensitivity, with symptoms often more pronounced in the morning due to corneal edema [7].

FED usually manifests later in life, most commonly after the fourth decade, although earlier-onset forms have been associated with specific genetic mutations, such as *COL8A2*, while transcription factor 4 (TCF4) has been linked to late-onset disease [8-11]. Diagnosis is primarily based on slit-lamp examination, which identifies guttae, although additional imaging modalities, such as Scheimpflug tomography, optical coherence tomography, or pachymetry, may assist in detecting early corneal edema and monitoring disease progression [12].

Most patients with FED experience significant visual improvement following keratoplasty [13]. Although both PK and EK can provide good final visual outcomes, EK offers faster visual rehabilitation due to better preservation of corneal anatomy [14]. Over time, refinements in EK techniques have focused on optimizing graft thickness and surgical handling to further improve outcomes. In this context, advances in EK techniques have led to the development of UT-DSAEK and DMEK, which aim to improve visual outcomes while maintaining graft stability [15-17].

Despite the increasing use of DMEK and UT-DSAEK, evidence comparing these two techniques specifically in patients with FED remains limited and heterogeneous. Variations in graft thickness definitions, surgical techniques, and study design have contributed to inconsistent findings across studies. Considering the growing global prevalence of FED and the increasing demand for endothelial transplantation procedures [18,19], a clearer understanding of the comparative outcomes of these surgical approaches is clinically important.

Therefore, this systematic review and meta-analysis aimed to compare the postoperative clinical outcomes of UT-DSAEK and DMEK in adult patients with FED.

## Review

Methodology

The purpose of this systematic review and meta-analysis is to contrast the postoperative clinical outcomes of DMEK and UT-DSAEK for the surgical treatment of adults with FED.

Search Method

**Figure 1:**
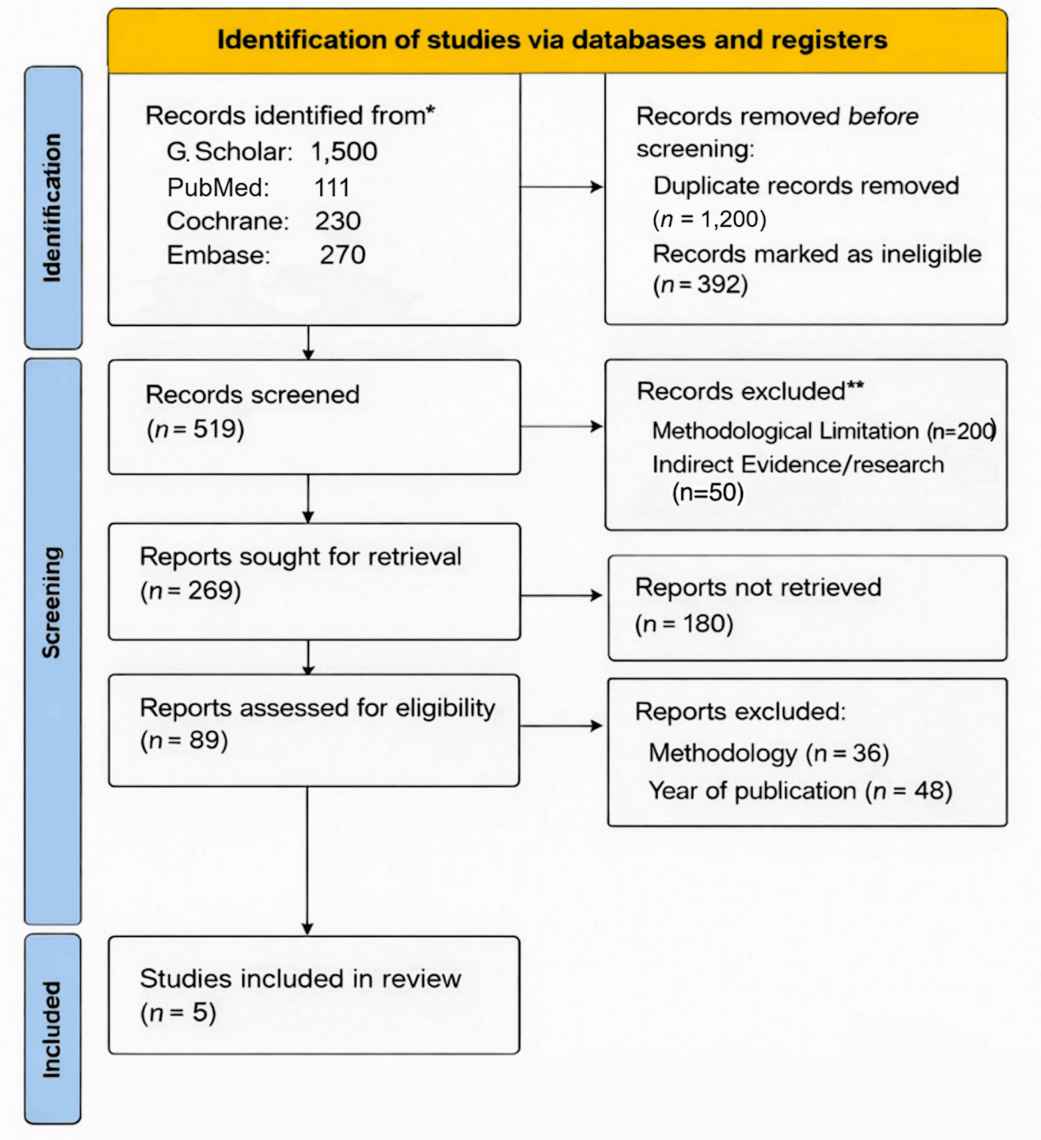
PRISMA chart * Search conducted with Google Scholar (G. Scholar) **Ineligible by date of publication, publication type, and study design PRISMA: Preferred Reporting Items for Systematic Reviews and Meta-Analyses

Our literature search was conducted through peer-reviewed electronic databases such as PubMed (MEDLINE), Cochrane Library, Embase, and Google Scholar, last run on May 1, 2024, and followed the Preferred Reporting Items for Systematic Reviews and Meta-Analyses (PRISMA) standards (Figure 1) [20]. The search combined controlled vocabulary terms and free-text keywords using Boolean operators (AND/OR). The core search string included combinations such as (“Descemet membrane endothelial keratoplasty” OR “DMEK”) AND (“ultrathin Descemet stripping automated endothelial keratoplasty” OR “UT-DSAEK”) AND (“Fuchs endothelial dystrophy” OR “FED”). Additionally, the possible studies were manually identified from the reference lists of the obtained articles. The following databases were thoroughly searched for RCTs and non-randomized comparable clinical trials. Only peer-reviewed full-text studies published in English within the last ten years were included. Non-English publications were excluded due to feasibility constraints, and conference abstracts were not considered because they often lack complete outcome data and detailed methodology, which may introduce reporting bias.

Eligibility Criteria and PICOS Scheme

We analyzed randomized controlled trials (RCTs) and non-randomized comparable studies (NRSs) with a paired contralateral-eye design that includes a comparison between DMEK and UT-DSAEK for treating people with FED.

The PICOS scheme was created with the following factors: (P) adults with FED; (I) DMEK; (C) UT-DSAEK; (O) visual and safety outcomes; (S) RCTs and comparable NRSs. Using ROBINS-I, we ranked the bias risk of NRSs [21].

Inclusion criteria: Adults with a clinical diagnosis of FED requiring corneal transplantation surgery were included in the study. UT-DSAEK has been reported in the literature to have a graft thickness of 50-130 μm [22]. Patients who were not pseudophakic before transplantation, receiving phaco-DMEK/UT-DSAEK, combining cataract surgery with corneal transplantation, were also included.

Exclusion criteria: Studies with patients with visually significant comorbidities were disregarded (e.g., glaucoma, previous corneal surgeries, trauma, visually significant macular or optic nerve pathology, glaucoma drainage devices, aphakia, anterior chamber intraocular lenses, scleral-fixated intraocular lenses, and uveitis). There were no limitations on age or gender.

The primary outcome was best-corrected visual acuity (BCVA) in logMAR at 12 months. The secondary outcomes included BCVA at six months, ECD at 12 months, re-bubbling, overall postoperative complications, graft failure, graft rejection, and intraocular pressure elevation. BCVA was defined as the efficacy outcome, while ECD and complication rates were considered safety outcomes.

Data Extraction

Study selection and data extraction were performed independently by two reviewers. Any discrepancies were resolved through discussion and consensus. Among the 2111 studies examined, 89 were relevant and evaluated for quality and outcomes to gather data. Five studies (three RCTs and two NRSs) met our inclusion criteria. The following data were gathered from each of the included studies: the first author's name, the year of publication, the overall number of patients (N) who were enrolled in each study, the BCVA at six and 12 months, the rate of ECD loss, the total number of complications of each study, and the percentage of re-bubbling, respectively. To measure effects, weighted mean differences (WMDs) and risk ratios (RRs) were used. This meta-analysis did not require either a patient's informed consent or authorization from an ethical review board because all the data it contained were taken from previously published literature.

Data Analysis

The extracted data encompassed the principal methodological and clinical characteristics of the included studies, as summarized in Table 1. For each study, we documented the design (RCT or NRS) and the level at which randomization or outcome reporting was conducted (participant- or eye-based). Participant characteristics included the total number of enrolled individuals, the corresponding number of eyes analyzed, sex distribution, and mean age. Additionally, we recorded the specific intervention and comparator evaluated in each study, the predefined primary and secondary outcome measures, and the duration of follow-up. This structured data extraction ensured consistency across studies and facilitated meaningful comparison of their methodological features and clinical findings.

**Table 1:** Design and demographics of studies on outcomes following DMEK and UT-DSAEK RCT = Randomized controlled trial; NRS = non-randomized study; N/A = not available; BCVA = best-corrected visual acuity; FED = Fuchs endothelial dystrophy; UT-DSAEK = ultrathin Descemet stripping automated endothelial keratoplasty; DMEK = Descemet membrane endothelial keratoplasty; CS = contrast sensitivity; logMAR = logarithm of the minimum angle of resolution

Study	Design	Participants		Age (years)	Eyes (number)	BCVA (logMAR)		Postoperative month	p-value	Conclusion
		Male	Female			UT-DSAEK	DMEK			
Matsou (United Kingdom), 2021 [23]	RCT	24	33	54–91	56	0.11±0.09	0.04±0.13	12	≤0.02	Better BCVA after DMEK at 12 months in eyes with FED
Dunker (Netherlands), 2020 [24]	RCT	N/A	N/A	68–74	54	0.15±0.11	0.18±0.14	12	0.06	BCVA higher after DMEK
Chamberlain (United States of America), 2019 [15]	RCT	37	13	50–72	50	0.16±0.18	0.04±0.12	12	<0.001	Better BCVA after DMEK at 3, 6, and 12 months; similar complication rates
Mencucci (Italy), 2020 [25]	NRS	2	16	73.5±7.93	36	0.10±0.04	0.07±0.07	12	0.24	No difference in BCVA; Better CS, posterior corneal aberrations, and patient satisfaction after DMEK
Torras-Sanvicens (Spain), 2021 [26]	NRS	4	6	75.40±6.69	20	0.16±0.14	0.21±0.29	6	0.99	No differences in terms of objective and subjective visual quality

It is impossible to mask the surgeons performing the procedure. If participants or assessors were not blinded to the intervention, studies were found to have a high risk of performance and detection bias [27].

We intended to apply ROBINS-I to estimate the possibility of bias in NRSs that met our inclusion criteria [28]. Using this tool, we would develop a fictional, generic target randomized trial that accounts for the population, the intervention, the comparator, and the expected outcomes. The conceptualization of this hypothetical target trial enables the research question to be precisely defined. In addition, it allows for identifying potential complications regarding the equipment employed to measure an outcome domain or the time of measurements.

We designated DMEK as the experimental intervention and UT-DSAEK as the control intervention while utilizing the ROBINS-I tool. In our hypothetical generic goal trial, the outcome that results from randomly allocating participants to either DMEK or UT-DSAEK at baseline is what we are interested in. Confounding domains in NRSs that fulfill the requirements for inclusion may indicate whether a patient will receive the experimental or control intervention. The varying follow-up intervals between eyes are the confounding factor that poses the most risk. Although UT-DSAEK is a more recent intervention, many patients in NRSs had received UT-DSAEK more recently than DMEK. The surgeries could have been separated by months or even years, which could have thrown the results off and not led to long-term results.

Ocular comorbidity is the second domain that can confuse. In more complex cases where the patient has aphakia or an anterior chamber lens implanted or has had prior glaucoma filtering surgery, DSAEK may be utilized more frequently than DMEK since it is thought to be technically simpler [5]. This is why positioning a DMEK graft is fragile and more challenging than the one used in DSAEK [5]. Despite all this, no large studies describe a proportional superiority of UT-DSAEK in similar ocular comorbidities, like DSAEK. Therefore, our review's inclusion criteria excluded patients with prior ocular comorbidities.

Systematic review

The systematic review comparing these two surgical techniques helped collect all the necessary data to carry out the statistical analysis according to the abovementioned methodology [29]. Then, to present the results of the review, a summary table is created that includes the studies and their categorization in terms of population, primary outcomes, results, etc. (Table 1). Additionally, Table 2 presents the studies' data analysis and baseline characteristics.

**Table 2:** Preoperative BCVA, ECD, and CCT UT-DSAEK = Ultrathin Descemet stripping automated endothelial keratoplasty; DMEK = Descemet membrane endothelial keratoplasty; BCVA: best-corrected visual acuity; ECD = endothelial cell count; CCT = central corneal thickness; N/A = not specified; logMAR = logarithm of the minimum angle of resolution

Study	Preop. BCVA (logMAR)		Preop. ECD (cells/mm^2^)		Preop. CCT (μm)	
	DMEK	UT-DSAEK	DMEK	UT-DSAEK	DMEK	UT-DSAEK
Matsou (United Kingdom), 2021 [23]	0.38±0.15	0.38±0.23	2682±201	2632±160	N/A	N/A
Dunker (Netherlands), 2020 [24]	0.37±0.18	0.31±0.13	2679±157	2633±158	N/A	N/A
Chamberlain (United States of America), 2019 [15]	0.34±0.29	0.27±0.21	2771±150	2796±238	608±52	610±44
Mencucci (Italy), 2020 [25]	0.51±0.11	0.60±0.29	2626±124	2700±59	629±9.8	618±39
Torras-Sanvicens (Spain), 2021 [26]	0.43±0.17	0.49±0.22	2670±195	2520±257	656.28±39	671.12±61

Statistical analysis

We used the STATA software package to perform a statistical analysis (StataCorp. 2013. Stata Statistical Software: Release 13.0. College Station, TX: StataCorp LLC). For BCVA measured in logMAR, a continuous and clinically interpretable measure, we calculated the WMD and 95% confidence intervals (CIs). For ECD, where measurement scales varied across studies, we calculated the WMD and 95% CIs. For postoperative complications, re-bubbling rate, and IOP rise among patients undergoing UT-DSAEK versus DMEK, we calculated the RR and 95% CIs. The level of significance was set at 0.05, with the corresponding 95% CIs reported. Heterogeneity among studies was assessed using Cochran’s Q and I² statistics. A random-effects model was applied when considerable heterogeneity was detected (I² > 75%), consistent with conventional interpretations of high heterogeneity thresholds. Sensitivity analyses were conducted to evaluate the influence of individual studies on pooled results. Due to the small number of included studies per outcome (<10), formal assessment of publication bias using funnel plots, Begg’s rank correlation, and Egger’s regression tests was not performed, as these methods lack sufficient power and may yield misleading conclusions in small samples. A p-value <0.05 was considered statistically significant.

Only RCTs and paired contralateral-eye non-randomized studies were included in the quantitative synthesis. In contralateral-eye designs, each eye served as an internal control, thereby reducing between-patient confounding. The unit of analysis was the eye, and pooling was performed using the published eye-level summary statistics reported in the primary studies. Although eyes from the same patient are not statistically independent, most included studies did not report paired variance estimates or intraclass correlation coefficients that would allow adjustment for within-patient clustering. Therefore, eyes were analyzed as independent observations for meta-analytic purposes. This approach may lead to underestimation of variance and is acknowledged as a potential methodological limitation. Meta-regression analysis was conducted to examine the modifying effect of certain variables in the presence of significant heterogeneity.

Results

Study Selection

In total, 2111 articles were identified initially by searching through electronic databases. Subsequently, 1592 unrelated articles were removed by analyzing the titles and abstracts. For a thorough analysis, 519 papers were evaluated, and 250 articles were excluded after a full-text examination and independent review. Of them, 180 did not present usable data. Hence, five studies (three RCTs and two NRSs) were selected for this meta-analysis since they met our inclusion criteria.

Characteristics of the Included Studies and Quality Assessment

The range of research periods was between 2012 and 2024. The mean BCVA level and standard deviation values in patients with UT-DSAEK and DMEK were provided in each article. A total of 216 eyes were recruited in this meta-analysis; 110 eyes (50.93%) were enrolled in the DMEK group, and 106 eyes (49.07%) were enrolled in the UT-DSAEK group. The methodological quality of all included articles was evaluated using the ROBINS-I tool and GRADE (Grading of Recommendations Assessment, Development, and Evaluation) for NRSs (Tables 3-6).

**Table 3:** Risk of bias

Study	Risk of confounding	Bias in the selection of participants	Bias in the classification of intervention	Bias due to departures from intended intervention	Bias due to missing data	Bias in measurements of outcomes	Bias in the selection of the reported result
Matsou (United Kingdom), 2021 [23]	Serious Risk	Low Risk	Low Risk	Low Risk	Low Risk	Low Risk	Low Risk
Dunker (Netherlands), ±2020 [24]	Serious Risk	Low Risk	Low Risk	Low Risk	Low Risk	Low Risk	Low Risk
Chamberlain (United States of America), 2019 [15]	Serious Risk	Low Risk	Low Risk	Low Risk	Low Risk	Low Risk	Low Risk
Mencucci (Italy), 2020 [25]	Serious Risk	Low Risk	Low Risk	Moderate Risk	Low Risk	Low Risk	Low Risk
Torras-Sanvicens (Spain), 2021 [26]	Serious Risk	Low Risk	Low Risk	Low Risk	Serious Risk	Moderate Risk	Serious Risk

**Table 4:** ROBINS-I assessment tool of risk of bias. Study: Mencucci et al. (2020) [25]—paired contralateral-eye study ROBINS-I = Risk of bias in non-randomized studies of interventions; RCT = randomized controlled trial; NRS = non-randomized study; N/A = not available; BCVA = best-corrected visual acuity; FED = Fuchs endothelial dystrophy; UT-DSAEK = ultrathin Descemet stripping automated endothelial keratoplasty; DMEK = Descemet membrane endothelial keratoplasty; CS = contrast sensitivity; logMAR = logarithm of the minimum angle of resolution; ECD = endothelial cell density; IOL = intraocular lens

Robins-I domain	Risk of bias	Description
Bias due to confounding	Serious Risk	Patients underwent UT-DSAEK in one eye and, after an average of 6 months (6,3 ± 1,2 months), DMEK in their fellow eye. All patients were pseudophakic (spherical monofocal hydrophobic acrylic IOL, SA60AT, Alcon, Fort Worth, Texas, USA)
Bias in the selection of participants	Low Risk	Intervention and follow-up typically began simultaneously. Since UT-DSAEK was employed in one eye and DMEK in the other in all patients, there is no proof that they were chosen for the study based on factors measured after the intervention.
Bias in the classification of intervention	Low Risk	Well-defined surgical procedures
Bias due to deviation from the intended intervention	Moderate Risk	The surgeon was not so experienced with the DMEK technique. However, they highlighted that when he became more confident, he performed the DMEK surgeries. There is no evidence of differences in co-interventions.
Bias due to missing data	Low risk	No missing data reported. They mentioned that three patients were excluded (one due to serious post-op complication and two because they experienced severe visual loss due to age macular degeneration)
Bias in measurement of outcomes	Low risk	Retrospective study. BCVA measured in logMAR. ECD (Perseus, CSO, Italy)
Bias in selection of the reporting result	Low risk	No possibility of selective reporting for our pre-specified results
Overall risk	Serious risk	-

**Table 5:** ROBINS-I assessment tool of risk of bias. Study: Torras-Sanvicens et al. (2021) [26]—cross-sectional comparative and observational case series study ROBINS-I = Risk of bias in non-randomized studies of interventions; UT-DSAEK = ultrathin Descemet stripping automated endothelial keratoplasty; DMEK = Descemet membrane endothelial keratoplasty; BCVA = best-corrected visual acuity; ECD = endothelial cell density; logMAR = logarithm of the minimum angle of resolution; AMD = age-related macular degeneration

Robins-I domain	Risk of bias	Description
Bias due to confounding	Serious Risk	The most important bias is that the eye operated on in the first place was always UT-DSAEK, and the second eye undergoing DMEK was performed knowing the result of the first eye. Another confounding factor is the fact that two eyes in the UT-DSAEK group and no patient in the DMEK group underwent cataract surgery at the same time.
Bias in the selection of participants	Low Risk	There is no proof that they were chosen for the study based on factors measured after the intervention.
Bias in the classification of intervention	Low Risk	Well-defined surgical procedures
Bias due to deviation from the intended intervention	Moderate Risk	There is no information about the surgeon's training experience. Two eyes in the UT-DSAEK group and no patients in the DMEK group underwent cataract surgery at the same time.
Bias due to missing data	Serious Risk	No missing data reported. They mentioned one patient was excluded by age-related macular degeneration in the UT-DSAEK group and two patients by graft failure due to low ECD.
Bias in measurement of outcomes	Moderate Risk	Cross-sectional, comparative, and observational case series. BCVA converted logMAR and ECD (Corneal Specular Microscope SP (Topcon Medical Systems, Tokyo, Japan))
Bias in selection of the reporting result	Serious Risk	-

**Table 6:** GRADE assessment table UT-DSAEK = Ultrathin Descemet stripping automated endothelial keratoplasty; DMEK = Descemet membrane endothelial keratoplasty; BCVA = best-corrected visual acuity; ECD = endothelial cell density; logMAR = logarithm of the minimum angle of resolution; RR = risk ratio; MD = mean difference; GRADE: Grading of Recommendations Assessment, Development, and Evaluation

Outcome	No. of studies	Effect estimate (95% CI)	Direction	Certainty	Justification
BCVA at 6 months	5	MD = 0.586 logMAR (0.35–0.95)	Favors DMEK	Moderate (⬤⬤⬤◯)	Downgraded for risk of bias and slight inconsistency.
ECD at 6–12 months	5	MD = -253.6 cells/mm² (-409 to -98)	Favors UT-DSAEK	Low (⬤⬤◯◯)	Downgraded for heterogeneity and study quality.
Overall complications	4	RR = 0.64 (0.43–0.96)	Fewer in UT-DSAEK	Moderate (⬤⬤⬤◯)	Downgraded due to design limitations and low event numbers.
Re-bubbling	3	RR = 0.28 (0.10–0.82)	Fewer in UT-DSAEK	Moderate (⬤⬤⬤◯)	Downgraded for imprecision and limited sample size.

BCVA Postoperatively

**Figure 2:**
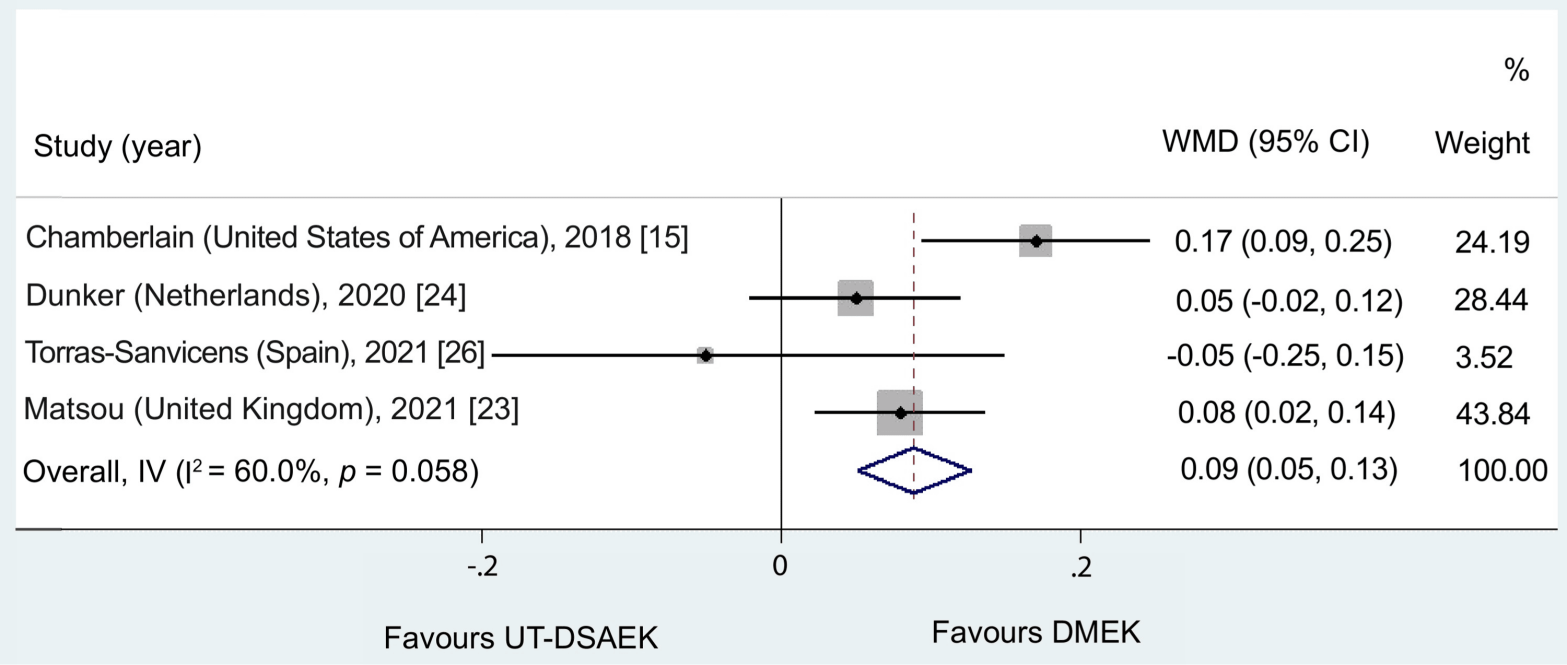
Results of the random-effects meta-analysis comparing the effects of DMEK and UT-DSAEK on best-corrected visual acuity (BCVA) at six months UT-DSAEK = Ultrathin Descemet stripping automated endothelial keratoplasty; DMEK = Descemet membrane endothelial keratoplasty; WMD = weighted mean difference; CI = confidence interval

Using a fixed-effects meta-analysis model, there was a statistically significant difference in BCVA between the UT-DSAEK and DMEK groups at six months postoperatively (WMD = 0.09, 95% CI = 0.05 to 0.13, p < 0.001). Lower logMAR values indicate better visual acuity; therefore, the positive WMD indicates superior visual outcomes in the DMEK group. According to the meta-analysis of the four included studies, Figure 2 shows that the WMD was equivalent to 0.089. Non-significant heterogeneity was detected when all studies were included (I^2^ = 60.0%, p = 0.06).

**Figure 3:**
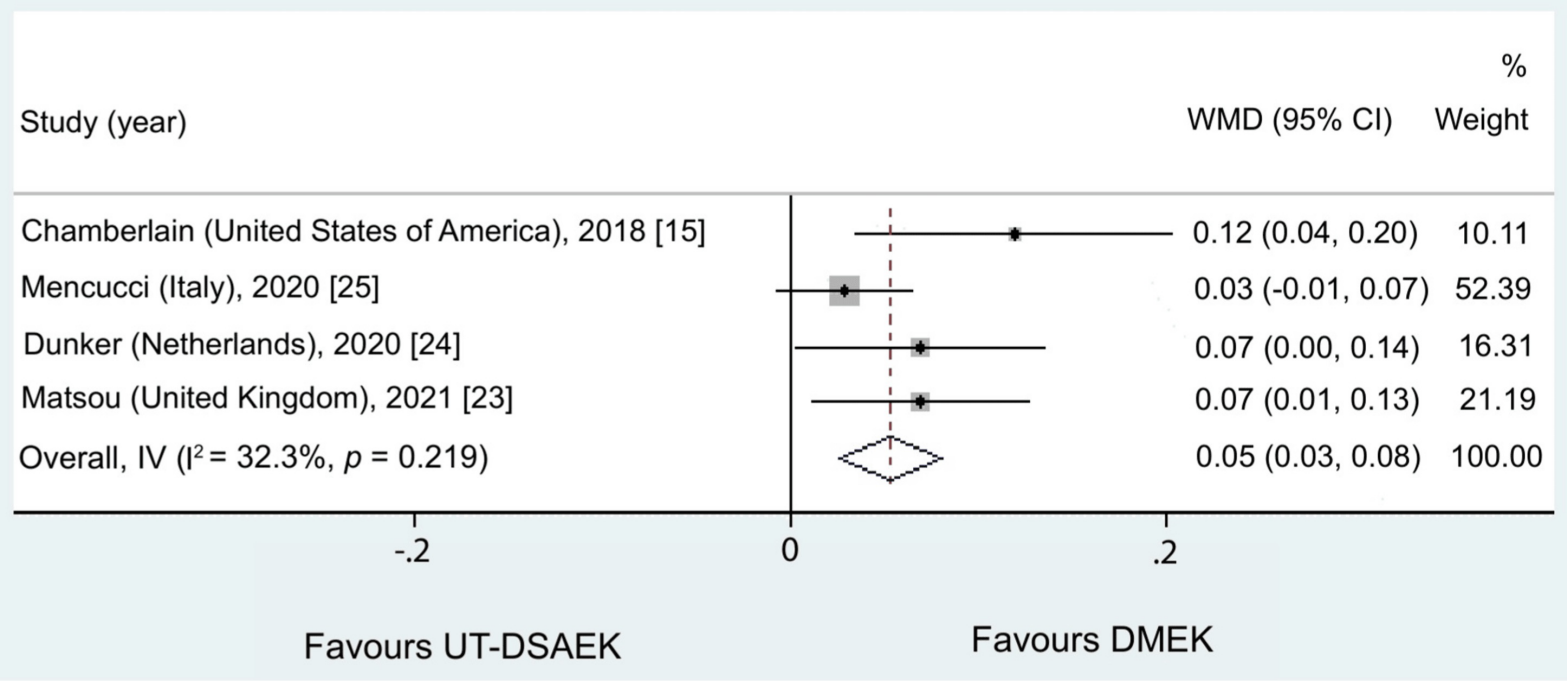
Results of the meta-analysis comparing the effects of DMEK and UT-DSAEK on best-corrected visual acuity (BCVA) at 12 months UT-DSAEK = Ultrathin Descemet stripping automated endothelial keratoplasty; DMEK = Descemet membrane endothelial keratoplasty; WMD = weighted mean difference; CI = confidence interval

At 12 months, DMEK demonstrated superior BCVA. In addition, non-significant heterogeneity was detected (I^2 ^= 32.3%, p = 0.22), and the fixed-effect model showed a WMD equal to 0.05 in favor of DMEK (95% CI = 0.03 to 0.08, p < 0.001). The overall fixed effect and heterogeneity of the pooled studies are summarized in Figure 3.

ECD Postoperatively

**Figure 4:**
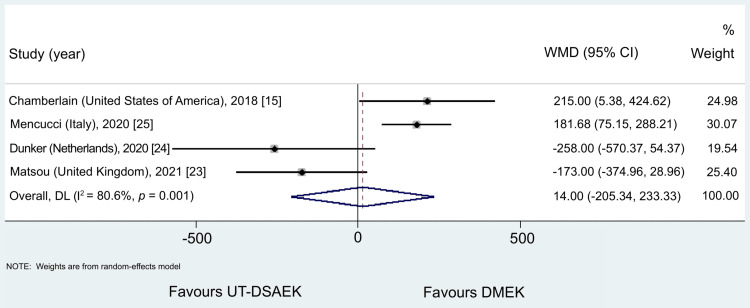
Results of the meta-analysis comparing UT-DSAEK and DMEK in terms of endothelial cell density (ECD) at 12 months UT-DSAEK = Ultrathin Descemet stripping automated endothelial keratoplasty; DMEK = Descemet membrane endothelial keratoplasty; WMD = weighted mean difference; CI = confidence interval

Four studies demonstrated ECD results in one year. There was no statistical difference between the two surgical techniques in the ECD in 12 months (WMD = 13.99, 95% CI = -205.336 to 233.330, p = 0.900). However, significant heterogeneity was detected between the four studies (I^2^ = 83.9%, p < 0.001), so a random-effect model was used (Figure 4).

Univariable meta-regression analyses were conducted to assess the modifying effects of study design, number of surgeons, year of publication, sample size, and geographic region on the effect size. None of the variables were statistically significant.

Postoperative Complications

**Figure 5:**
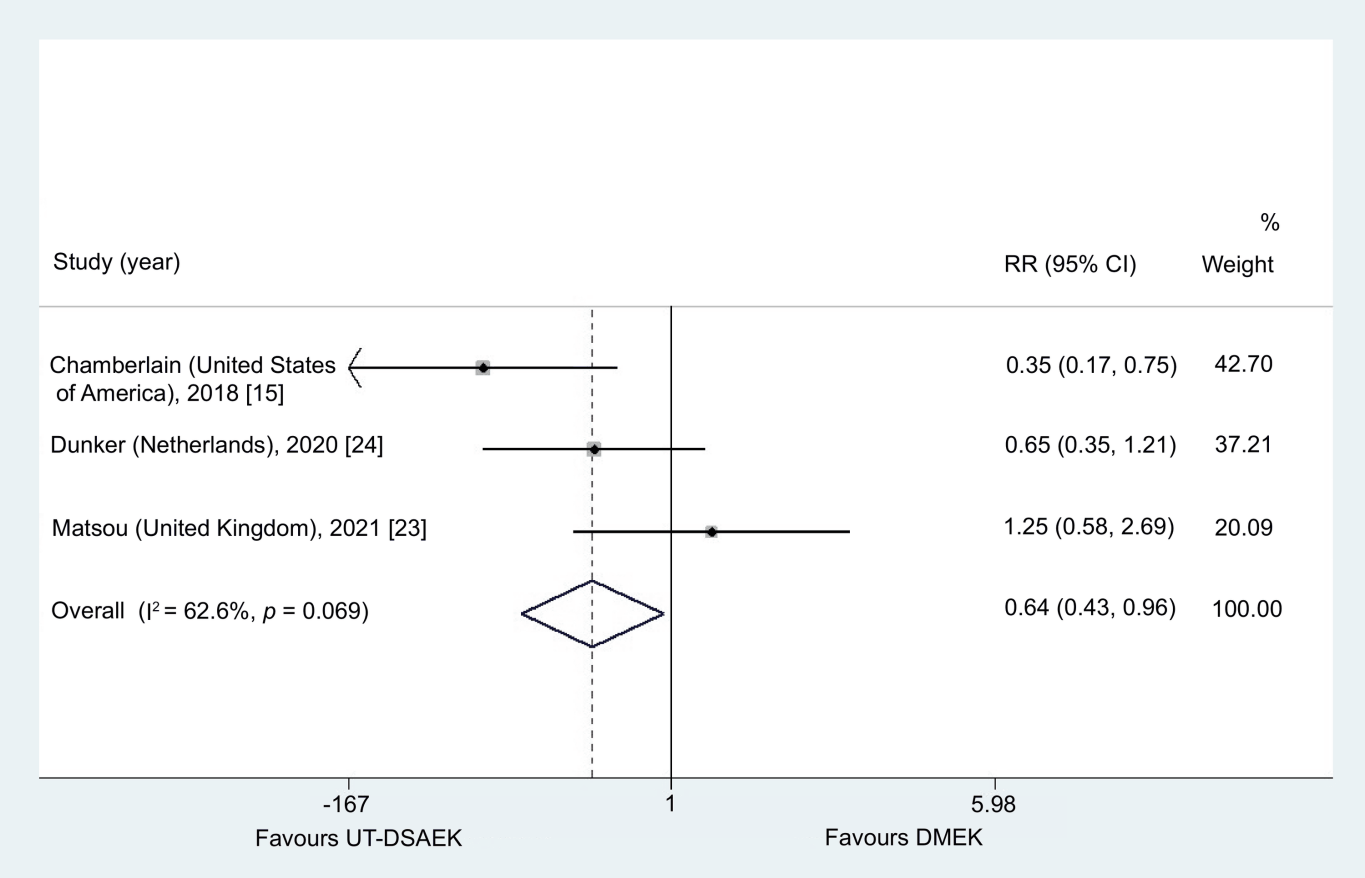
Results from the meta-analysis concerning postoperative complications between DMEK and UT-DSAEK UT-DSAEK = Ultrathin Descemet stripping automated endothelial keratoplasty; DMEK = Descemet membrane endothelial keratoplasty; RR = risk ratio; CI = confidence interval

UT-DSAEK was associated with significantly lower total complications (RR = 0.64, 95% CI = 0.43-0.96, p = 0.029). Non-significant heterogeneity was detected (I^2^ = 62.6%, p = 0.07) (Figure 5). One study reported one case of donor preparation failure for both the DMEK and UT-DSAEK groups [24]. According to the same study, three DMEK cases required re-transplantation [24]. Two studies reported two cases of graft failure in the DMEK group and one case in the UT-DSAEK group [15,23]. According to a fixed-effects model, the overall risk of adverse events was 0.64 times higher in the DMEK group.

Re-bubbling

**Figure 6:**
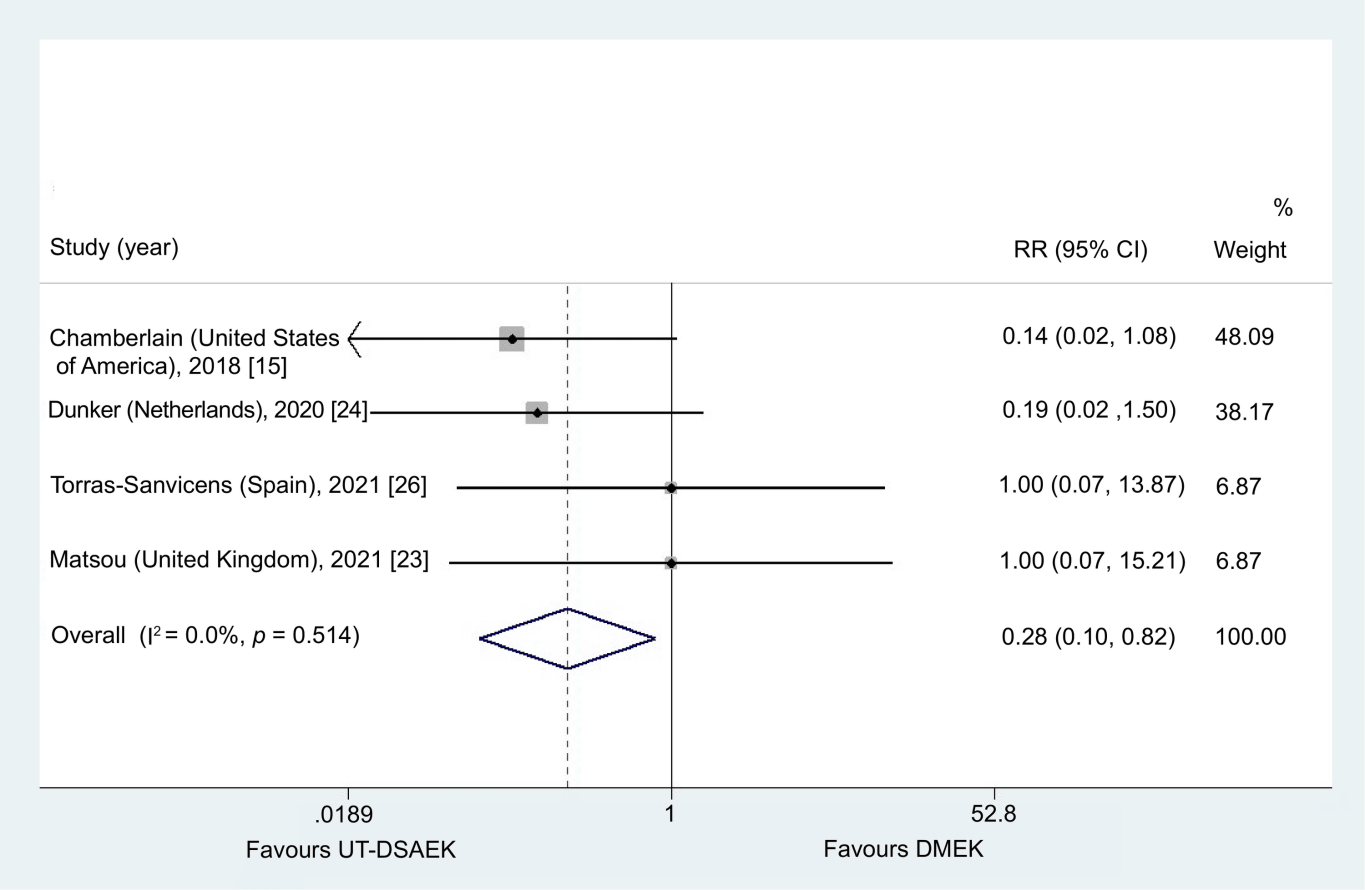
Conclusions of the meta-analysis about the possibility of re-bubbling UT-DSAEK = Ultrathin Descemet stripping automated endothelial keratoplasty; DMEK = Descemet membrane endothelial keratoplasty; RR = risk ratio; CI = confidence interval

The need for re-bubbling was significantly more frequent in the DMEK group (RR = 0.28, 95% CI = 0.10-0.82; p = 0.02). During the evaluation of the four studies, non-significant heterogeneity was identified (I^2^ = 0.0%, p = 0.51) [15,23,24,26]. Figure 6 summarizes the overall fixed effect and heterogeneity of the pooled studies.

IOP Postoperatively

**Figure 7:**
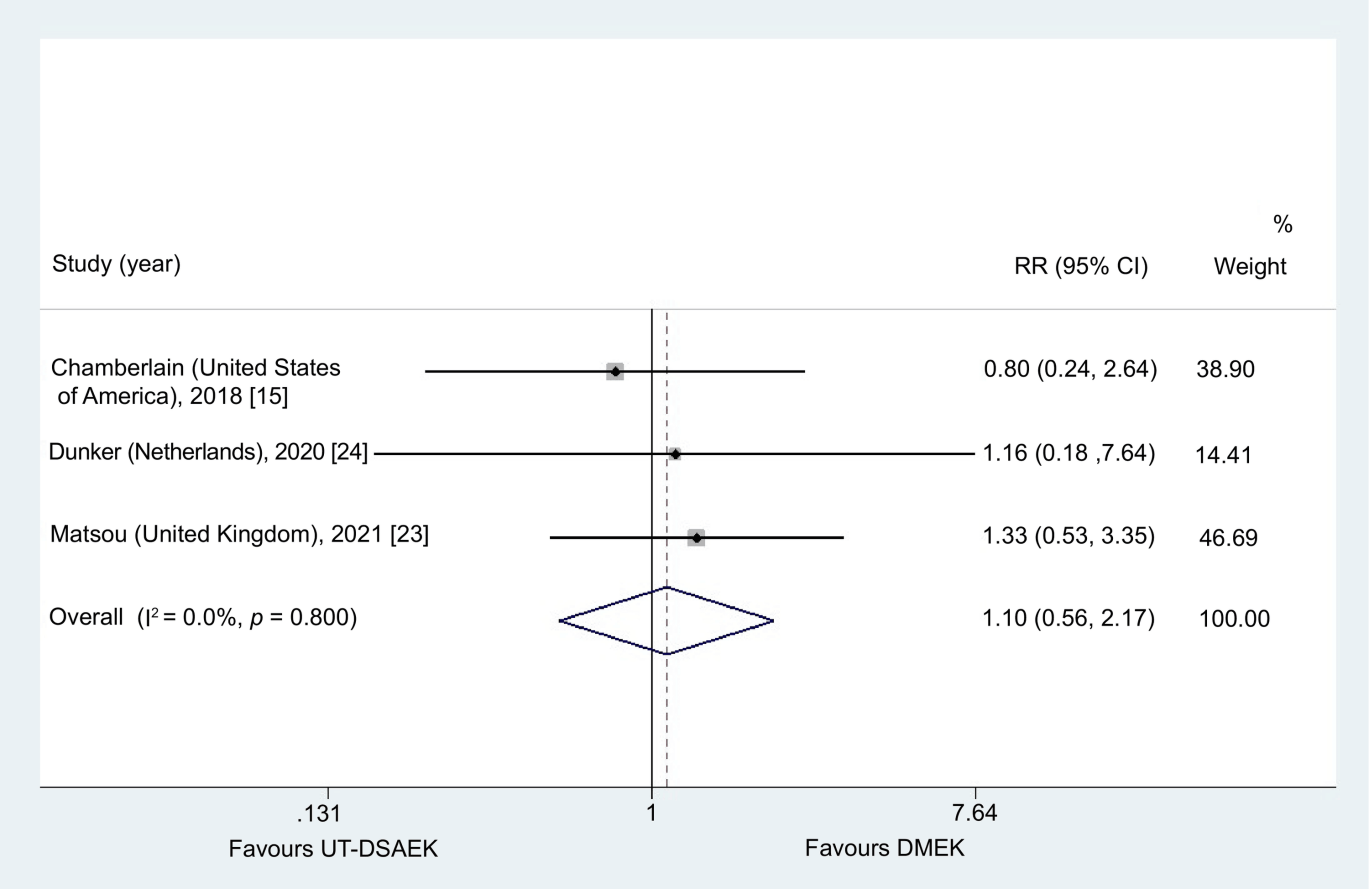
Results from the meta-analysis concerning the postoperative risk of intraocular pressure (IOP) elevation UT-DSAEK = Ultrathin Descemet stripping automated endothelial keratoplasty; DMEK = Descemet membrane endothelial keratoplasty; RR = risk ratio; CI = confidence interval

Only three studies examined the occurrence of postoperative IOP elevation [15,23,24]. No significant difference was observed in postoperative ECD at 12 months (RR = 1.10, 95% CI = 0.56-2.16; p = 0.78). Non-significant heterogeneity was detected (I^2^ = 0.0%, p = 0.800) (Figure 7).

Re-transplantation and graft rejection were documented in some cases that were successfully treated [15,23,24]. Also, there have been a few reports of patients who experienced serious vision loss as a result of other ophthalmologic comorbidities that worsened postoperatively, unrelated to the intervention; these patients were not counted in the final results [25]. One study included two eyes with pseudophakic bullous keratopathy (PBK) [15].

Risk of Bias

Table 3 displays the risk of bias for each domain and study, while Table 4 and Table 5 indicate the risk estimation for the two NRSs using the ROBINS-I tool. Risk-of-bias assessment was conducted independently by two reviewers using the ROBINS-I tool, with consensus reached through discussion.

Several non-randomized studies were judged to have a serious risk of bias, primarily due to potential confounding related to sequential contralateral-eye surgery and differences in follow-up timing between eyes. However, other domains, including outcome measurement, participant selection, and missing data, were generally rated as low risk. Considering the internal control nature of the contralateral-eye design and the overall methodological profile, the overall risk of bias across studies was judged to be moderate, despite serious confounding concerns related to sequential surgery.

Due to the small number of included studies per outcome (<10), formal assessment of publication bias using funnel plots and Egger’s/Begg’s tests was not performed, as funnel plot symmetry in small samples is unreliable and does not reliably indicate absence of bias [21].

Certainty of Evidence (GRADE)

The GRADE framework shown in Table 6 was applied to evaluate the certainty of evidence across different outcomes, including a specific assessment of publication bias among its five main domains. While ROBINS-I assesses internal validity at the individual study level, GRADE incorporates additional domains, including inconsistency, imprecision, and publication bias, to determine overall certainty of evidence. This distinction explains why certain outcomes were rated as moderate certainty despite the presence of serious domains within the ROBINS-I assessment.

GRADE was selected as the method for assessing the risk of publication bias in this meta-analysis due to its transparency and established approach.

Discussion

We conducted a meta-analysis comparing UT-DSAEK and DMEK in patients with FED. Both techniques significantly improved postoperative visual acuity. However, DMEK demonstrated slightly better visual outcomes, whereas UT-DSAEK was associated with fewer postoperative complications and lower re-bubbling rates. No significant differences were observed between the two techniques regarding ECD loss or postoperative IOP elevation. These findings highlight an important clinical trade-off between superior visual outcomes with DMEK and greater procedural stability with UT-DSAEK.

Visual Outcomes

Visual recovery after EK is influenced by several factors, including patient characteristics, graft thickness, interface quality, and corneal optical properties [30-37]. DMEK restores corneal anatomy more closely to its physiological structure because it transplants only the Descemet membrane and endothelium, without stromal tissue [31,32]. In contrast, UT-DSAEK includes a thin stromal layer that may contribute to subtle optical irregularities affecting postoperative vision.

Progressively thinner UT-DSAEK grafts have been associated with improved visual outcomes, although the relationship between graft thickness and postoperative acuity remains debated [38-41]. A meta-analysis by Beal et al. [42] reported favorable visual outcomes in grafts ≤130 μm without demonstrating a clear thickness-acuity relationship. Accordingly, UT-DSAEK was defined as graft thickness ≤130 μm in our analysis, and the RCT by Matsou et al. [23] was included. Previous comparative studies have reported heterogeneous findings: some favor DMEK for visual acuity [15,43], whereas others report similar outcomes between DMEK and ultrathin or nanothin DSAEK techniques [44,45].

DMEK has gained increasing popularity for the treatment of endothelial dysfunction due to faster visual recovery and lower rejection rates compared with DSAEK [14]. In FED, the most common indication for EK, combined procedures with cataract surgery are frequently performed [46]. In our meta-analysis, most patients were pseudophakic or underwent combined procedures, resulting in comparable visual acuity outcomes at one year. Similarly, Mukhija et al. [47] reported greater BCVA improvement at one year following triple DMEK, although earlier outcomes were comparable.

Assessment of visual outcomes may also vary depending on the measurement methods used. ETDRS visual acuity measurements may fluctuate even in the absence of clinical change [48], while early visual symptoms in FED may relate more strongly to contrast sensitivity than to BCVA alone [49]. Consequently, functional visual parameters may complement conventional acuity measurements when evaluating surgical outcomes.

Clinical Applicability and Surgical Expertise

Beyond visual outcomes, practical considerations may influence the choice between UT-DSAEK and DMEK. Graft preparation techniques and surgical logistics may affect procedural efficiency and postoperative outcomes. Pagano et al. [50] reported lower graft detachment rates when grafts were prepared by the surgeon rather than the eye bank, while Romano et al. [51] demonstrated improved adhesion and lower re-bubbling rates with surgeon-prepared DMEK grafts. Economic considerations may also influence surgical decision-making, as locally prepared grafts have been reported to be more cost-effective in some healthcare settings [52], and cost-effectiveness analyses have suggested that DSAEK-based procedures may remain preferable in certain contexts despite the visual advantages of thinner graft techniques [53].

The increasing adoption of DMEK has contributed to changing patterns in endothelial keratoplasty practice. Data from the Eye Bank Association of America indicate a progressive rise in DMEK procedures since 2013, while the number of DSAEK and DMEK procedures performed in recent years remains comparable [54,55].

Surgical expertise also plays a significant role in determining outcomes. DMEK is technically more demanding than UT-DSAEK, and several studies suggest improved surgical results after approximately 25-30 procedures, reflecting the impact of the learning curve [56]. In contralateral-eye studies, DMEK was frequently performed after UT-DSAEK in the same patient, which may introduce temporal bias favoring the later procedure. Nevertheless, once the learning phase is surpassed, outcomes tend to become more consistent. In settings with limited DMEK experience or in patients with complex anterior segment anatomy, UT-DSAEK may offer more predictable and reproducible results [15,57-59].

Safety and Postoperative Complications

Although DMEK demonstrated superior visual acuity outcomes, it was associated with a higher risk of graft detachment requiring re-bubbling. Various strategies, such as the use of SF6 anterior chamber tamponade, have been proposed to reduce detachment risk [15,60], although some studies have reported no significant effect on re-bubbling rates, ECD, or visual outcomes [61,62].

Severe vision-threatening complications were rare across studies, and graft rejection or failure occurred infrequently. Long-term data suggest lower rejection rates following DMEK compared with DSAEK, although follow-up in the included studies was limited [63,64]. Importantly, our analysis demonstrated significantly lower rates of graft detachment, re-bubbling (RR = 0.28, 95% CI = 0.10-0.82), and overall complications (RR = 0.64, 95% CI = 0.43-0.96) in the UT-DSAEK group [23,24,26].

Elevated intraocular pressure was the second most common postoperative complication and occurred at similar rates in both groups. This complication is typically related to steroid response or pupillary block and can usually be managed through peripheral iridectomy or corticosteroid adjustment [65,66].

Future Research

Further high-quality RCTs with larger sample sizes are needed to better define the comparative effectiveness of UT-DSAEK and DMEK in patients with FED. A recent meta-analysis by Maier et al. [67] compared these techniques in patients with both FED and PBK, but it was published after the predefined literature search deadline of our study. PBK may arise from several causes, including endothelial trauma from intraocular surgery, viral endotheliitis, trauma, or exfoliation syndrome [68], and its corneal morphology differs from that observed in FED [69]. For this reason, our analysis focused exclusively on FED patients.

The prevalence of FED is expected to increase as global populations age [18]. Consequently, the demand for EK procedures is likely to continue rising. Our findings suggest that DMEK may provide superior visual outcomes, whereas UT-DSAEK offers lower complication rates and greater surgical stability. Therefore, the choice between these techniques should be individualized according to patient characteristics, surgeon experience, and available surgical resources. In clinical practice, DMEK may be preferred when performed by experienced surgeons seeking optimal visual outcomes, whereas UT-DSAEK remains a valuable and safer alternative in complex cases or in settings with limited surgical expertise or constrained healthcare resources.

Potential Biases in the Review Process

Only non-randomized studies with a paired contralateral-eye design were included, as this design allows comparison within the same patient and helps reduce between-patient confounding. Although such studies have inherent limitations, they are generally less prone to unmeasured confounding than unmatched observational designs. However, the moderate risk of bias across the included studies lowers the overall certainty of the findings and suggests that results should be interpreted with caution. In addition, the small number of included studies and limited sample sizes may have reduced statistical power for certain outcomes, meaning that non-significant findings, such as for IOP or re-bubbling, may reflect imprecision rather than a true absence of effect. Reported conflicts of interest were minimal, with only one study disclosing external funding from the National Eye Institute and Research to Prevent Blindness [15].

Characteristics of the Excluded Studies

We discovered three recent systematic reviews and meta-analyses contrasting DMEK with UT-DSAEK for corneal endothelial dysfunction that were all published after May 1, 2024 (the date of our last recent literature search), even though they all included patients with FED.

We excluded the following research: (1) Duggan et al. [35]: They demonstrated findings of higher-order aberrations of the anterior and posterior cornea at six and 12 months after DMEK and UT-DSAEK. However, their participants had PBK or FED. (2) Ang et al. [70]: They compared DMEK with UT-DSAEK for BCVA and vision-related quality of life, but they included patients with PBK. (3) Kurji et al. [45]: They conducted a prospective comparative case series to compare nanothin DSAEK with DMEK for patients with FED. However, it was not a paired contralateral-eye study. (4) Tourabaly et al. [44]: They compared visual outcomes after EK (DMEK, UT-DSAEK, nanothin DSAEK, conventional DSAEK), but they also included patients with FED, PBK, and herpes endotheliitis. (5) Romano et al. [51]: They compared the clinical results and consequences of preloaded DMEK with preloaded UT-DSAEK in patients with FED and PBK. (6) Rose-Nussbaumer et al. [60]: They discussed the DETECT trial's 24-month postoperative results. The visual acuity of the six- or 12-month follow-up was not highlighted. (7) Machalińska et al. [71]: It was not a paired contralateral eye study, and they also included patients with FED and PBK. (8) Maier et al. [67]: It is a great meta-analysis, including patients with PBK and FED.

Quality of the Evidence

Only three RCTs to date have compared thinner DSAEK and DMEK at one year, and only one recently released trial has examined the outcomes of the two methods at two-year follow-up [15,23,24,60]. In the NRSs we chose, DMEK followed UT-DSAEK in every patient, which may have caused an imbalance in unidentified confounders, and the certainty of the evidence was very low in terms of visual outcomes. Furthermore, after a six-month period had passed since the first eye's UT-DSAEK procedure, surgeons specifically performed DMEK on the contralateral eye. Therefore, despite all trials reporting balanced baseline BCVA, we downgraded this area to moderate risk of bias.

Despite variations in preoperative endothelial cell densities across studies, factors such as simultaneous cataract extraction performed as a triple procedure in phakic patients might be responsible for the heterogeneity in final ECD values.

Given that this is a major limitation of this meta-analysis, it is important to investigate further the possibility of bias resulting from UT-DSAEK being performed before DMEK in all patients. Also, each surgeon often performs DMEK in small groups of patients, and in the intervals between both operations, they would continue their surgical training. Consequently, it is likely that even other similar small case series or these non-randomized cohort studies that make up this review will most closely match modern clinical practice. Finally, we agree that as a surgeon acquires more experience, the necessity for further repositioning (re-bubbling) may diminish.

None of the study participants experienced severe eyesight loss following surgery. However, these studies enrolled a limited number of patients to accurately measure this result. With relatively few graft rejections and graft failures, almost everyone who participated in the research had good graft survival.

Limitations

Our meta-analysis included one study reporting two eyes with PBK [15]. Although this represents a small proportion of the total sample and is unlikely to have materially influenced the pooled estimates, it remains a limitation. Variability in UT-DSAEK graft characteristics and surgical techniques across studies may have affected outcome comparability. In addition, demographic data were not uniformly reported in detail across trials. Differences in ECD findings were observed, despite relatively low overall heterogeneity in visual acuity outcomes, complications, re-bubbling rates, and IOP elevation. Another limitation is that the systematic review protocol was not prospectively registered in an international database such as PROSPERO. Although the review followed PRISMA guidelines and a predefined search strategy, the absence of prospective registration may increase the risk of reporting bias.

Non-English publications were excluded, introducing potential language bias. Furthermore, follow-up was limited to one year, restricting assessment of long-term graft survival and complications. The literature search was conducted up to May 1, 2024, according to the predefined protocol, and additional real-world data or longer follow-up studies may further refine comparative estimates between DMEK and UT-DSAEK.

## Conclusions

To sum up, compared to UT-DSAEK, DMEK showed better visual outcomes regarding overall visual acuity. Both techniques are still effective options for a cornea specialist regarding FED patients. To better understand the differences between the two surgical techniques, additional large multicenter RCTs are needed to provide results in terms of postoperative BCVA, endothelial cell loss, complication rates, and graft survival for patients with FED. Determining whether the surgical technique would be the gold standard for FED patients or providing the best postoperative outcomes is crucial for corneal surgeons.
